# Editorial: Recent advances in skin disorders: from basic mechanisms to therapies

**DOI:** 10.3389/fcell.2024.1379183

**Published:** 2024-02-28

**Authors:** Floriana Cappiello, Han Liu

**Affiliations:** ^1^ Laboratory Affiliated to Pasteur Italia-Fondazione Cenci Bolognetti, Department of Biochemical Sciences, Sapienza University of Rome, Rome, Italy; ^2^ Ben May Department for Cancer Research, The University of Chicago, Chicago, IL, United States

**Keywords:** skin disorder, wound healing, inflammatory dermatoses, skin cancer, peptide

## Introduction

As the first line of defense against the outside environment, skin is the largest organ of our body and is susceptible to various external stressors, such as UV irradiation, microorganism infections, or mechanical injuries (Fuchs, 2008). These external factors, mingled with internal factors, trigger the perturbation of skin homeostasis and result in a series of cutaneous disorders such as chronic wounds, psoriasis, and tumors. Therefore, a deep understanding about pathological mechanisms of skin disorders helps to advance the cutaneous biology field and future clinical applications. In this Research Topic, we present a collection of original research and review articles with the aim to focus on the most recent advances of the underlying mechanisms of different skin disorders and the development of therapeutic strategies to target them.

## Wound healing

Impaired wound healing always poses a great challenge to both the patients and the healthcare system. Peptides have been the most prevalent and biologically important class of compounds used for wound healing. Their major advantages include low production costs and sale prices. Many animal derived peptides have been characterized and show promising ability on wound healing. In this Research Topic, a mini review article by Wang et al. summarizes amphibian-derived wound-healing-promoting peptides, mainly identified from anuran (frogs and toads) amphibians. These peptides, abundantly expressed in the skin, allow the animal better adapt to the harsh environmental conditions. The authors also compare the mechanisms of these peptides. In particular, they mention that many of these molecules can also be classified into antimicrobial peptides (AMPs) and suggest that AMPs from frogs can be used as a reservoir for screening wound-healing-promoting peptides.

Many wound healing promoting peptides have been identified during the last decades. These natural amino acid-based therapeutic peptides have two intrinsic drawbacks: membrane impermeability and poor *in vivo* stability, which represent major stumbling blocks for peptide drug development. Cyclization is a common peptide modification which increases proteolytic stability and cell-permeability and allows mimicking and stabilization of the peptide secondary structure (Sohrabi et al., 2020). In this Research Topic, another review article by Lee et al. provides an overview of cyclic peptides that have been shown to promote wound healing as well as recovery and tissue injury prevention. The authors discuss current state of the clinically applications of cyclic peptides, and provide an assessment including both advantages and challenges in incorporating cyclic peptides into clinical care.

## Skin cancer

Melanoma is the most aggressive type of skin cancer and develops from the melanocytes, the cells of the epidermis that are responsible for the pigmentation of the skin. Both melanoma and melanocyte regeneration at its early stages share activation of cell proliferation and migration (Belote et al., 2021). However, because of its abnormal proliferation, invasive growth, and metastasis, melanoma significantly differs from the regenerating melanocytes at their later stages where they stop proliferating and differentiate. In an original research article, Katkat et al. compare the gene expression profile of melanocyte regeneration and nevi/melanoma and uncover the opposite regulation of a substantial number of genes related to Wnt signaling and transforming growth factor beta (TGF-β)/(bone morphogenetic protein) BMP signaling pathways between regeneration and cancer. Functional activation of canonical Wnt or TGF-β/BMP pathways enhanced melanocyte regeneration but potently suppressed the invasiveness, migration, and proliferation of human melanoma cells *in vitro* and *in vivo*. Characterizing signaling pathways differentially regulated between regeneration and cancer could be exploited to block tumor growth and develop new anti-cancer therapies.

Non-melanoma skin cancers, including epidermoid carcinoma, represent about 1/3 of all malignancies diagnosed yearly worldwide (Hu et al., 2022). The α7-type nicotinic acetylcholine receptor (α7-nAChR) plays a significant role in cancer pathogenesis promoting the growth and metastasis of solid tumors (Wang and Hu, 2018). The human secreted Ly6/uPAR-Related Protein 1 (SLURP-1) is a selective negative allosteric modulator of α7-nAChR, and recently, loop I of SLURP-1 has been identified as the active site responsible for the antitumor activity of the protein (Bychkov et al., 2021). In their original research article, Shlepova et al. studied the molecular mechanisms underlying the SLURP-1 antiproliferative activity in epidermoid carcinoma A431 cells. They also investigated the antitumor activity of SLURP-1 and synthetic 21 amino acids peptide mimicking its loop I (named “Oncotag”) in a xenograft mice model of epidermoid carcinoma. While both SLURP-1 and Oncotag inhibited tumor growth *in vivo*, only Oncotag selectively interacted with α7-nAChR and caused prolonged downregulation of pro-oncogenic signaling. This selective inhibition of α7-nAChRs by drugs, such as Oncotag, may be a promising strategy for cancer therapy.

## Inflammatory dermatoses

Several diseases with multiple etiologies, including genetic factors, infections, and immune dysregulation, are defined as inflammatory dermatoses. Recently, it has been reported that gut microbiota is closely associated with inflammatory dermatoses, such as psoriasis, rosacea, and atopic dermatitis, by affecting host skin immunity (Polkowska-Pruszyńska et al., 2020).

In their original research article, Mao et al. performed a bidirectional two-sample Mendelian randomization (MR) analysis study and identified 76 causal relationships between the gut microbiome and six inflammatory dermatoses. These results deepen the understanding of the “gut-skin axis” and may be helpful for the clinical prevention and treatment of inflammatory dermatoses by regulating the structure of the gut microbiota.

In conclusion, the findings and ideas presented in this Research Topic provides insights into various skin diseases such as skin tumor and impaired wound healing. We hope that this Research Topic will increase the interest for the underlying mechanisms of different skin disorders and the potential therapeutic strategies to target them.
